# Comparative Chloroplast Genome Analysis of Anchusa and the Adulterants of HERBA ANCHUSAE

**DOI:** 10.3390/genes17090993

**Published:** 2026-08-24

**Authors:** Liang Chen, Yong-Zhen Zhong, Xiao-Qin Xu, Yue-Shun Wu, Di-Na Mai, Yi Tong, Wei Lan

**Affiliations:** 1 State Key Laboratory Basis of Xinjiang Indigenous Medicinal Plants Resource Utilization, Xinjiang Technical Institute of Physics and Chemistry, Chinese Academy of Sciences, Urumqi 830011, China; 13579832835@163.com (L.C.); maidn@ms.xjb.ac.cn (D.-N.M.); 2 University of Chinese Academy of Sciences, Beijing 101408, China; 3 Xinjiang Key Laboratory of Traditional Chinese Medicine Concoction Research, Traditional Chinese Medicine Hospital Affiliated to Xinjiang Medical University, Urumqi 830000, China; 18437015289@163.com; 4 Key Laboratory of Chinese Medicinal Resource from Lingnan, Ministry of Education, School of Pharmaceutical Sciences, Guangzhou University of Chinese Medicine, Guangzhou 510006, China; 5 South China Botanical Garden, Chinese Academy of Sciences, Guangzhou 510650, China; 6 Xinjiang Key Laboratory of Chinese Materia Medica and Ethnic, Xinjiang Institute of Materia Medica, Urumqi 830000, China; xjxuxq@163.com; 7 College of Traditional Chinese Medicine, Xinjiang Medical University, Urumqi 830091, China

**Keywords:** *Anchusa*, *Echium vulgare*, chloroplast genome, molecular identification, Boraginaceae

## Abstract

Background: The genus *Anchusa* L. includes plants used in Uyghur medicine for their anti-inflammatory and analgesic effects. However, in China, the botanical origin of HERBA ANCHUSAE (Niushecao, a Uyghur medicinal herb) is severely confused. Traditional identification methods and standard DNA barcodes do not work well for these close relatives. Chloroplast genomes are known to contain variable regions that can help distinguish species, yet no such study has been done for *Anchusa*. Therefore, We compared the complete chloroplast genomes of six *Anchusa* species and the main adulterants of Niushecao. Methods: We analyzed genome structure, repeat sequences, codon usage bias, and nucleotide diversity (Pi), as well as conducted comparative and phylogenetic analyses. Results: All genomes shared a typical ring-shaped quadripartite structure, ranged from 150,178 to 150,844 bp in size, and contained the same set of genes. Despite this overall conservation, we identified several highly variable spots, mostly located in non-coding intergenic spacer regions. Using two complementary approaches—sliding window analysis and mVISTA-based sequence visualization—we identified three overlapping regions (*rbcL*-*psaI*, *petA*-*psbJ*, and *trnC-GCA*-*petN*) as candidate DNA barcodes for species identification. Our phylogenetic tree showed that *Anchusa strigosa* Banks & Sol. is most closely related to the true medicinal species *Anchusa azurea* Mill. (Bootstrap support (BS) = 100%), while other look-alikes formed separate branches. Conclusions: These findings provide the first chloroplast genomic resources for this genus and offer potential molecular markers for authenticating *Anchusa* medicinal materials, laying a foundation for future development of molecular authentication methods.

## 1. Introduction

*A. azurea* Mill. is widely distributed across the temperate regions of the Northern Hemisphere, but is absent from the Americas and East Asia. Its distribution extends from eastern Central Europe through the Mediterranean region to the Western Himalayas. It commonly inhabits wastelands, fallow fields, and roadsides, occurring from near sea level to low-altitude mountainous areas [1]. *A. azurea* has a long history of medicinal use. In Turkey, dried leaves are powdered and applied topically to dry wounds. Alternatively, the root is decocted and mixed with egg yolk and beeswax to prepare an ointment for wound healing [2,3]. In Iran, the leaves are used in decoctions to treat colds, sore throat, and chest pain [4]. Furthermore, *A. azurea* is used as a stimulant, tonic, and demulcent for treating bile disorders, fever, cough, and asthma, and also as a diuretic for bladder and kidney stones [5].

The Uyghur medicinal herb HERBA ANCHUSAE (Niushecao) is derived from the dried aerial parts of *A. azurea* [*Anchusa italica* Retz.] (Boraginaceae), known as “Gaozivan” in Uyghur. It is officially listed in the Drug Standard of the Ministry of Public Health of the People’s Republic of China (Uyghur Medicine Volume) [6]. Niushecao has a long history of medicinal use in the Xinjiang region of China. It is widely employed for treating dry-cold or black-bile disorders involving the nervous, psychiatric, respiratory, and cardiovascular systems. Specific conditions include dry brain deficiency, depression, cough, and asthma [7]. In Uyghur medicine, Niushecao is a commonly used clinical drug for cardiovascular, cerebrovascular, and respiratory diseases. It also serves as the principal ingredient in classical Uyghur compound preparations such as the Abnormal Black Bile Maturation Agent and Aifeitimeng Decoction [8,9]. Modern studies have demonstrated that Niushecao possesses a rich chemical composition, including volatile oils, flavonoids, tannins, phenolic acids, triterpenoids, and alkaloids. Among these, isorhamnetin and rosmarinic acid are considered key bioactive compounds [10,11]. Recent pharmacological investigations have confirmed a broad spectrum of activities. These include anti-inflammatory, analgesic, antitumor, antioxidant, antiviral, neuroprotective, endocrine-modulating, and cardiovascular protective effects [7,9,12,13].

*A. azurea* is used as an important ethnic medicine in Xinjiang. However, it is not native to China and is cultivated only on a limited scale in places like Kashgar. Consequently, Niushecao crude drug material is almost entirely imported, mainly from Pakistan [14,15]. Our previous market surveys and literature reviews have revealed serious misidentification of the original plant sources of domestic Niushecao. These problems can be summarized into three main aspects. First, a considerable portion of imported Niushecao material originates from *A. strigosa* Banks & Sol., not from *A. azurea*. No authentic *A. azurea* specimens were identified in our sampling. Second, the botanical origin of Niushecao varies among different regions in China. For example, the Chinese Materia Medica lists *Borago officinalis* L., a species from the same family [16]. Third, several other medicinal *Anchusa* species are easily confused with *A. azurea*, and some Boraginaceae species share highly similar macroscopic traits with Niushecao. Consequently, multiple adulterants are present on the market. Common adulterants include *A. strigosa*, *A. officinalis* L., *A. arvensis* (L.) M.Bieb., *A. capensis* Thunb., *A. undulata* L. subsp. *hybrida* (Ten.) Cout., *Echium vulgare* L., and *Cynoglossum amabile* Stapf & Drummond [15,17,18]. The circulation of these adulterants severely compromises the quality and clinical consistency of Niushecao. It also undermines the accuracy and reproducibility of chemical and pharmacological studies of this herb.

Accurate botanical identification of crude drugs is a prerequisite for chemical, pharmacological and clinical studies. Although some research has addressed the identification of Niushecao, most studies have relied on traditional methods. These methods include macroscopic authentication, microscopic examination, thin-layer chromatography, and quantitative chemical assays. These methods have clear limitations when applied to fragmented processed materials or to closely related species [15,18,19]. DNA-based molecular identification has become an important tool for authenticating Chinese herbal medicines, offering high accuracy, reproducibility, and independence of morphological features. To date, molecular identification of Niushecao has mainly relied on ITS2 sequences [15,18,20]. These sequences have been used to distinguish *A. strigosa*, *B. officinalis*, and other *Anchusa* species. However, ITS2 sequences provide limited phylogenetically informative sites. In addition, no systematic comparative chloroplast genome study has been conducted on *Anchusa.* Genomic data for this genus remain scarce. Therefore, further molecular identification of Niushecao and its adulterants is needed.

Plant organellar genomes, particularly chloroplast genomes, exhibit highly diverse architectures across the tree of life. Most land plant chloroplast genomes possess a highly conserved quadripartite circular structure. They typically range from 115 to 165 kb in length and consist of a large single-copy (LSC) region, a small single-copy (SSC) region, and a pair of inverted repeats (IRs) [21]. Despite this conserved framework, plastomes vary greatly among lineages in genome size, IR boundaries, gene content, repeat sequences, and substitution rates [21]. The synonymous substitution rate of plastomes is approximately half that of nuclear genomes. However, it can fluctuate dramatically among lineages. These variations are shaped by multiple mechanisms, including selection pressure, mutation burden, and localized hypermutation [21]. In addition, mitochondrial and plastid genomes have independently evolved many similar genomic features. However, mitochondrial genomes generally exhibit greater architectural complexity [22]. Chloroplast genomes offer notable advantages over nuclear and mitochondrial genomes for identifying plant-derived medicinal materials, due to their relatively small size, conserved structure, and moderate evolutionary rates. These features make them a key source of molecular markers. These markers are widely used in plant identification, evolutionary biology, and genetic diversity assessment [23]. Conventional DNA barcodes based on chloroplast genome sequences, such as *rbcL*, *matK*, and *psbA-trnH*, have been important tools for herbal medicine identification. They are universal, easy to apply, and have performed reliably in authenticating many medicinal materials, including PINELLIAE RHIZOMA [24] and SOPHORAE TONKINENSIS RADIX ET RHIZOMA [25]. However, for closely related, morphologically similar, or evolutionarily complex taxa, conventional barcodes often fail due to limited informative sites. To overcome this limitation, a comparative framework based on complete chloroplast genomes has been established and is now a key strategy for precise discrimination of closely related species. This approach has led to significant progress in several taxa. For example, in ASARI RADIX ET RHIZOMA, this approach revealed an atypical tripartite genome structure, resolved the phylogenetic relationships among *Asarum heterotropoides* Fr. Schmidt var. *mandshuricum* (Maxim.) Kitag., *Asarum sieboldii* Miq. f. *seoulense* (Nakai) C.Y. Cheng et C.S. Yang, and *A. sieboldii* Miq. In addition, it identified taxon-specific hypervariable regions [26]. In *Polygonatum* Mill., conventional barcodes have proven inadequate. This genus has experienced complex reticulate evolution. However, comparative chloroplast genome analysis successfully identified hypervariable regions. These regions can distinguish morphologically similar species, such as *Polygonatum sibiricum* Redouté and *P. cyrtonema* Hua [27]. However, no study has yet systematically applied the comparative chloroplast genome approach to identify the botanical origin of the medicinal genus *Anchusa*.

The genus *Anchusa* L. (Boraginaceae) contains approximately 35 species worldwide [28] (based on APG IV; https://www.mobot.org/MOBOT/research/APweb/, accessed on 14 March 2026). Eight of these species are documented as medicinal [29,30,31]. To date, no systematic comparative study of the *Anchusa* chloroplast genome has been conducted. In our study, we selected six *Anchusa* species that are morphologically similar and closely related to *A. azurea*, as well as several adulterants (including *E. vulgare*), for comparative chloroplast genome analysis. The aim of this study is to characterize structural variations among the six chloroplast genomes and to fill the gap in genomic data. We will identify hypervariable regions and recommend them as candidate DNA barcode loci. These regions may serve as potential markers for molecular authentication of Niushecao. Our findings will provide fundamental data and a theoretical reference that may facilitate the development of molecular identification tools.

## 2. Materials and Methods

### 2.1. Plant DNA Extraction and Sequencing

The sources of samples used for de novo sequencing and assembly are listed in Table 1. Specimens were identified as *A. strigosa* and *E. vulgare* by Yi Tong (Associate Professor, Guangzhou University of Chinese Medicine). Voucher specimens are deposited in the herbarium of Guangzhou University of Chinese Medicine (GUCM) under the accession numbers Liang C20251029 and Liang C20251021, respectively.

Total plant DNA was extracted by Verygenome Technology Co., Ltd. (Guangzhou, China). Sequencing libraries were prepared with an average insert size of 350 bp. Paired-end sequencing (150 bp) was performed on the DNBSEQ-T7 platform (MGI). The primer sequences used for library construction were as follows: forward primer, 5′-CTCTCAGTACGTCAGCAGTTNNNNNNNNNNCAACTCCTTGGCTCACAGAACGACATGGCTACGA-3′; reverse primer, 3′-CCGGTTCGCCAGAATCCTTCTGTTNNNNNNNNNNGACTATTCCAGCGGTACG-5′ (N represents a random nucleotide). Approximately 3 Gb of raw data were obtained per sample. The raw sequencing reads generated in this study have been deposited in the NCBI Sequence Read Archive (SRA) under the BioProject accession number PRJNA1502504. Raw data for *A. capensis* were downloaded from the SRA database. Raw data for *A. undulata* subsp. *hybrida*, *A. azurea*, and *A. officinalis* were downloaded from the ENA database (Table 2). Authentic *A. azurea* samples were not available for this study. The identity of the publicly available *A. azurea* sequences could not be independently verified. Quality control was performed using fastp v0.23.4 [32].

### 2.2. Chloroplast Genome Assembly, Annotation, and Physical Mapping

Complete chloroplast genomes of six Boraginaceae species were assembled de novo using GetOrganelle v1.7.7.0 [33], with k-mer values of 65, 105, and 127. Annotation was performed with the Plastid Genome Annotator (PGA) [34]. The reference genomes used were *N. vesicaria* (L.) Rchb. for *Anchusa* species and *Echium plantagineum* L. for *E. vulgare* (Table 2). The *A. arvensis* chloroplast genome (GenBank accession OZ374795.1) was re-annotated following the same procedure. Manual correction was performed using Geneious Prime 2025.0.2 [35]. Circular chloroplast genome maps were drawn using OGDRAW (https://chlorobox.mpimp-golm.mpg.de/OGDraw.html, accessed on 11 January 2026) [36].

### 2.3. Repeat Sequence Identification

REPuter [37] (https://bibiserv.cebitec.uni-bielefeld.de/reputer, accessed on 18 January 2026) was used to identify interspersed repeats (forward repeats, reverse repeats, complement repeats, and palindromic repeats). The minimum repeat unit length was set to ≥ 30 bp, with a Hamming distance of 3.

MISA-web [38] (http://pgrc.ipk-gatersleben.de/misa, accessed on 12 January 2026) was used to detect simple sequence repeats (SSRs). The detection criteria were: mononucleotide SSR unit ≥ 10, dinucleotide SSR unit ≥ 5, trinucleotide SSR unit ≥ 4, and tetra-, penta-, and hexanucleotide SSR unit ≥ 3.

ChiPlot (https://www.chiplot.online, accessed on 17 January 2026) was used for data visualization.

### 2.4. Codon Usage Bias

Protein-coding sequences (CDSs) shared by the six *Anchusa* species were extracted using PhyloSuite v2 [39] and then screened. Duplicate genes and genes shorter than 300 bp were removed. Only sequences containing standard bases (A, T, G, C) and with both a start codon (ATG) and a stop codon (TAG, TGA, or TAA) were retained. The relative synonymous codon usage (RSCU) was calculated using the RSCUcaller package (v1.0) [40] in R (v4.4.1). Statistical significance of differences among species was assessed using the Kruskal–Wallis test, followed by Dunn’s test. The compared unit was defined as per-codon RSCU values per species (64 codon-level observations per species). The final results were visualized using ggplot2 package [41]. RSCU heatmaps were generated using TBtools-II v2.390 [42]. An RSCU value greater than 1 indicates a higher usage frequency, whereas a value below 1 indicates a lower usage frequency [43]. Other data were visualized using ChiPlot (https://www.chiplot.online, accessed on 17 February 2026).

### 2.5. Comparative Sequence Analysis

Global alignment and sequence identity comparison of the six *Anchusa* chloroplast genomes were performed using mVISTA [44] in LAGAN mode, with *A. strigosa* as the reference, to visualize genomic differences. Inverted repeat (IR) boundary contraction and expansion were analyzed and visualized using IRscope [45] (https://irscope.shinyapps.io/irapp/, accessed on 28 December 2025) to generate simplified diagrams of the chloroplast genomes. Collinearity analysis was performed using the Mauve v1.1.3 plugin [46] in Geneious Prime to detect rearrangement and inversion events.

### 2.6. Sliding Window Analysis

Sliding window analysis of the six *Anchusa* chloroplast genome sequences was performed with DnaSP v6.0 [47] to identify hypervariable regions and calculate nucleotide diversity (Pi). Parameters were set with a step size of 200 bp and a window length of 600 bp. Fragments with Pi > 0.025 and a length of at least 150 bp were screened as hypervariable regions. Their genomic positions were determined based on the gene annotation results.

### 2.7. Phylogenetic Tree Construction

Three outgroup species, *H. arborescens* L., *E. strigosa* (Willd.) Diane & Hilger, and *T. montana* Lour., were downloaded from NCBI (Table 2). Maximum likelihood (ML) trees were constructed for *Anchusa*, nine closely related species and three outgroups using complete chloroplast genomes. An additional ML tree was constructed using shared CDSs extracted with PhyloSuite v2 [39]. Multiple sequence alignment was performed with MAFFT v7.490 [48], followed by trimming with trimAL v1.4 [49]. ML trees were constructed using IQ-TREE v1.6.12 [50] with 1000 bootstrap replicates to assess branch support. Tree visualization and annotation were constructed using the ChiPlot online platform [51].

## 3. Results

### 3.1. Assembly and Annotation Results

**Figure 1:**
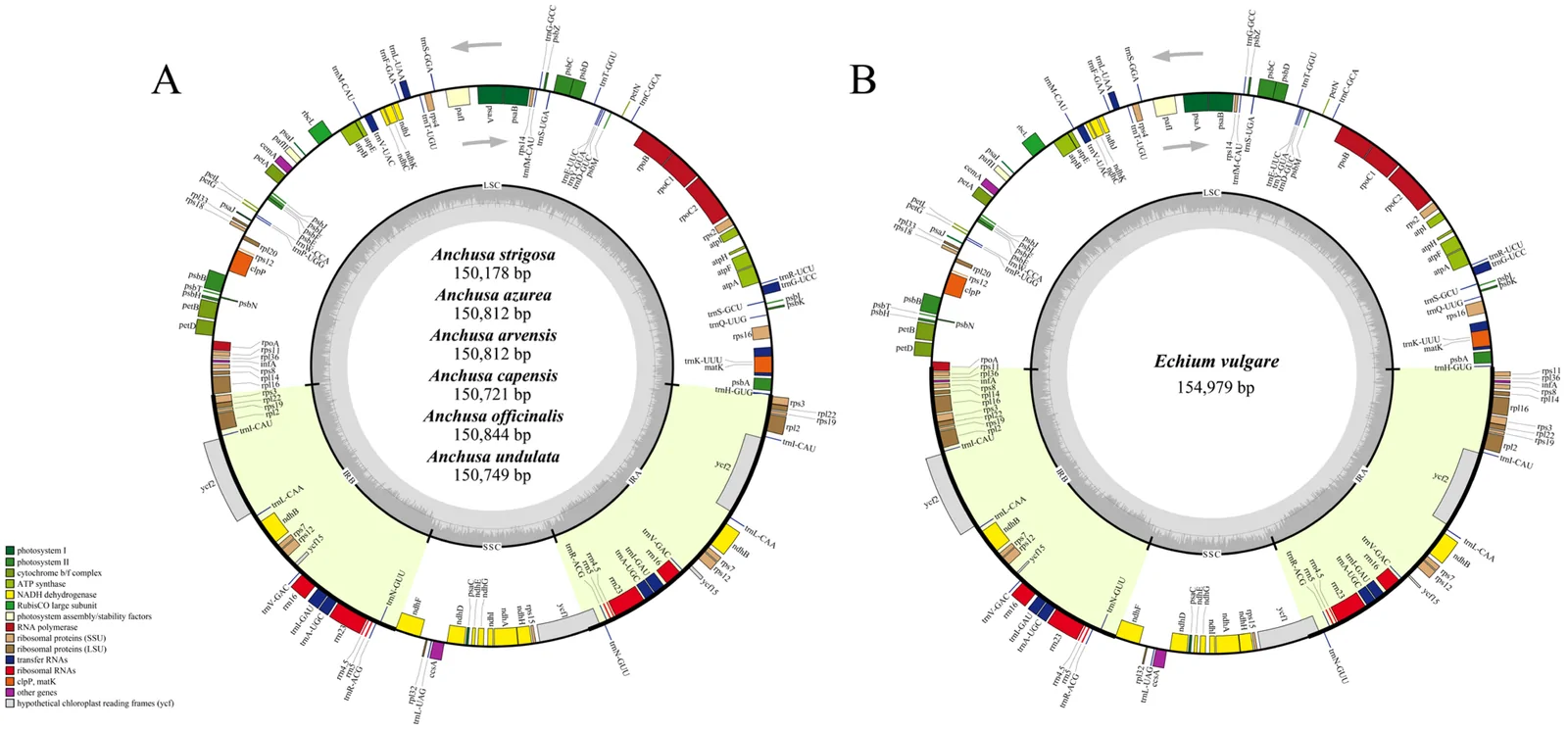
Physical maps of the complete chloroplast genomes of *Anchusa* species and *E. vulgare* L. (**A**) six *Anchusa* species; (**B**) *E. vulgare*.

Complete chloroplast genomes of six *Anchusa* species and the common adulterant *E. vulgare* were assembled and annotated. All genomes displayed the typical quadripartite circular structure (Figure 1). The total lengths of the six *Anchusa* chloroplast genomes ranged from 150,178 bp (*A. strigosa*) to 150,844 bp (*A. officinalis*) (Table 3). The LSC regions ranged from 79,780 to 79,976 bp. The SSC regions ranged from 16,782 to 16,871 bp. The IR regions ranged from 26,710 to 27,043 bp. Overall GC content was 37.9%, with IR regions consistently showing a GC content of 42.8%, higher than that of the LSC regions (35.9–36.0%) and SSC regions (31.7–31.8%).

The complete chloroplast genome of *E. vulgare* was 154,979 bp in length (Table 3). Its LSC, SSC and IR regions were 76,348 bp, 17,311 bp, and 30,660 bp, respectively. The overall GC content was 37.4%, and the IR GC content was 41.6%. Both values were higher than those of the LSC (35.6%) and SSC (31.1%) regions.

The chloroplast genomes of the six *Anchusa* species and three adulterants (*E. vulgare*, *B. officinalis*, *C. amabile*) were annotated, each with 112 genes (78 protein-coding genes, 30 tRNA genes, and 4 rRNA genes) (Table 4). Recent findings have led to updated annotations. The *ycf3* and *ycf4* genes encode photosystem I assembly factors [52]. They were annotated as *pafI* and *pafII*, respectively. The *psbN* gene encodes photosystem biogenesis factor 1. It was annotated as *pbf1* [53].

The complete chloroplast genome of *E. vulgare* (154,979 bp) was larger than those of the *Anchusa* species and the other two adulterants. Its IR region (30,660 bp) was markedly longer than that of *Anchusa* species (26,710–27,043 bp) and far exceeded that of the congeneric *E. plantagineum* (25,754 bp) (Table 3). This IR expansion placed *rpl16*, *rpl14*, *rps8*, *infA*, *rpl36*, and *rps11* within the IR region. These genes therefore occurred as duplicated copies. The chloroplast genome of *B. officinalis* (149,835 bp) was slightly smaller than those of *Anchusa* species. Its GC content was slightly lower across all regions. However, its overall structure, gene types, and copy numbers were largely consistent with those of *Anchusa*. The *C. amabile* chloroplast genome (151,532 bp) was larger than those of *Anchusa* species. It had a notably extended LSC region (82,902 bp) and a markedly shortened IR region (25,632 bp). In contrast to *Anchusa*, the *C. amabile* chloroplast genome contained only single copies of *rps3*, *rpl22*, and *rps19*.

### 3.2. Repeat Sequence Analysis

**Figure 2:**
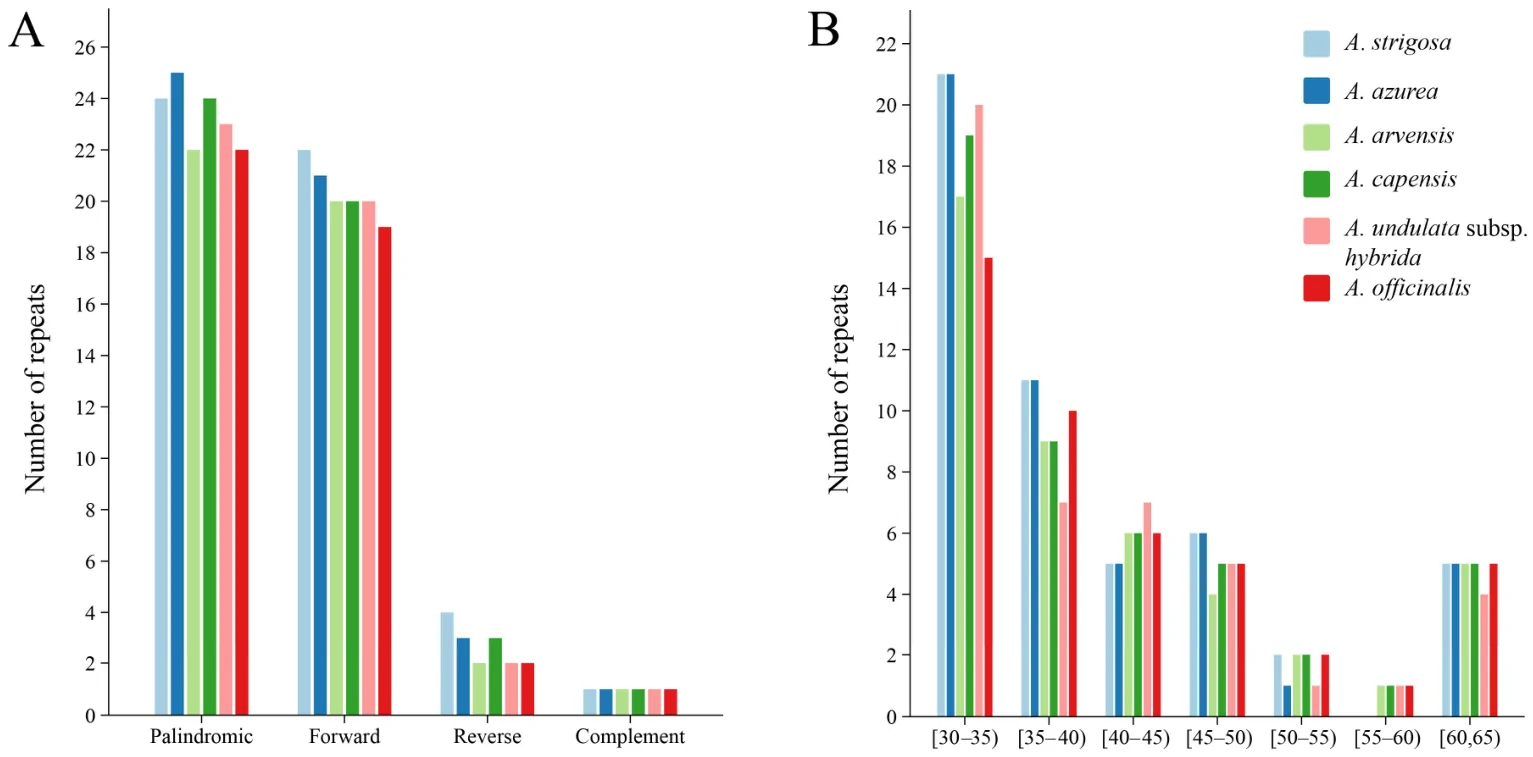
The types and existence of interspersed repeats in the chloroplast genomes of 6 species of *Anchusa*. (**A**) Different types of interspersed repeats in each chloroplast genome. (**B**) Numbers of different lengths of interspersed repeats.

Interspersed repeat analysis identified 44 (*A. officinalis*) to 51 (*A. strigosa*) repeats in the chloroplast genomes of the six *Anchusa* species (Figure 2A). Four types of repeats were detected: forward, reverse, complement, and palindromic. Palindromic repeats were dominant, ranging from 22 (*A. arvensis* and *A. officinalis*) to 25 (*A. azurea*). Forward repeats were the second most abundant, ranging from 19 (*A. officinalis*) to 22 (*A. strigosa*) (Figure 2A). Reverse and complement repeats occurred at low frequencies. Reverse repeats ranged from 2 (*A. arvensis*, *A. undulata* subsp. *hybrida*, *A. officinalis*) to 4 (*A. strigosa*). Complement repeats were present as a single copy in each species (Figure 2A). Notably, interspersed repeats of 30–35 bp were the most abundant. No repeat exceeding 64 bp was detected. Repeats of 55–60 bp were absent in *A. strigosa* and *A. azurea* (Figure 2B).

**Figure 3:**
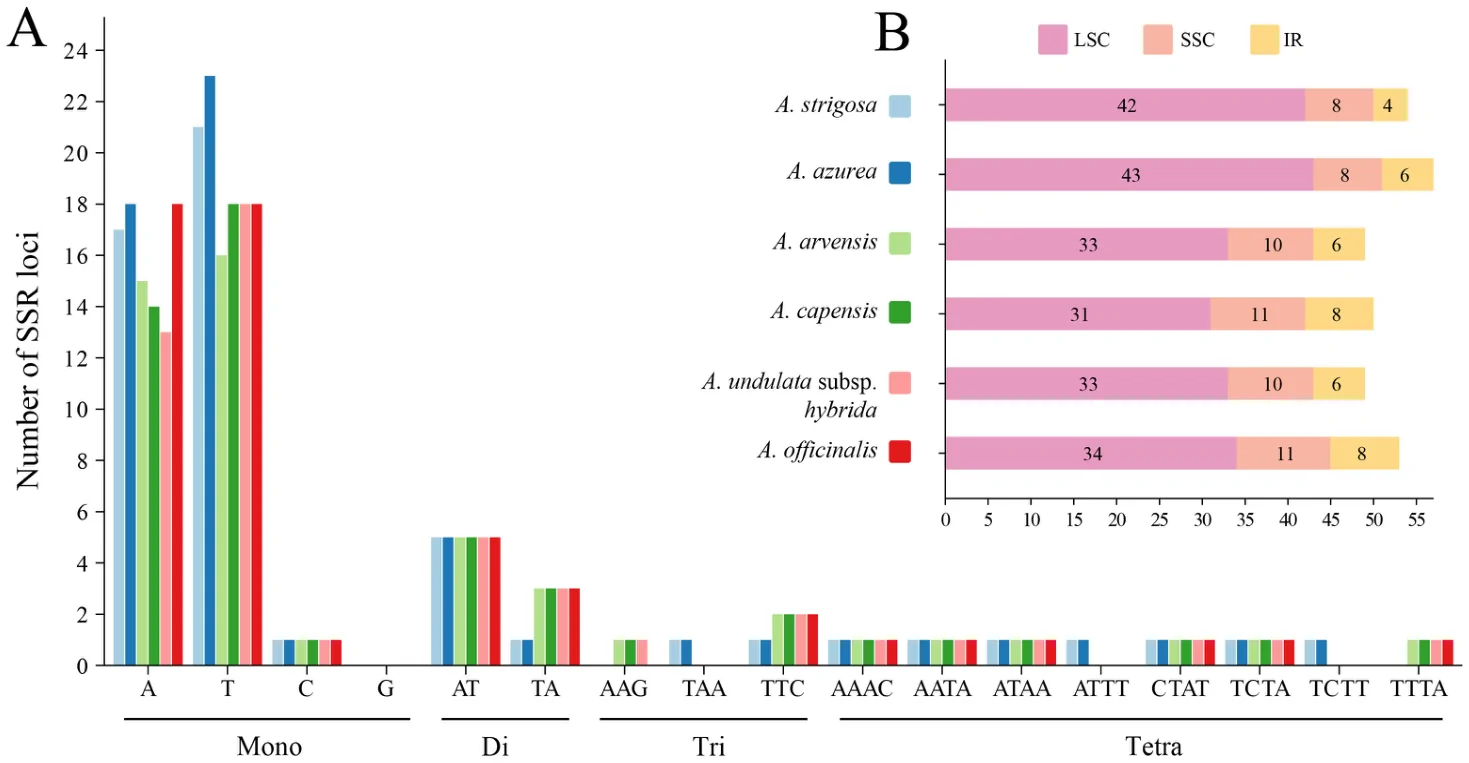
Chloroplast genome SSR analysis of *Anchusa* species. (**A**) Distribution of SSR loci across different nucleotide repeat types; (**B**) Distribution of SSR loci in different regions of the chloroplast genome.

MISA analysis detected 312 SSR loci across the six *Anchusa* chloroplast genomes (Figure 3A,B). These included four types: mononucleotide, dinucleotide, trinucleotide, and tetranucleotide repeats. Mononucleotide SSRs were the most abundant across all species (215 loci). Dinucleotide and tetranucleotide repeats had 44 and 38 loci, respectively. Trinucleotide repeats were the least frequent (15). Monomorphic A/T repeats dominated, accounting for 65.31–73.68% of all SSR loci. *A. azurea* contained the highest number of SSRs (57), while *A. arvensis* and *A. undulata* subsp. *hybrida* contained the fewest (49 each).

### 3.3. Codon Usage Bias Analysis

**Figure 4:**
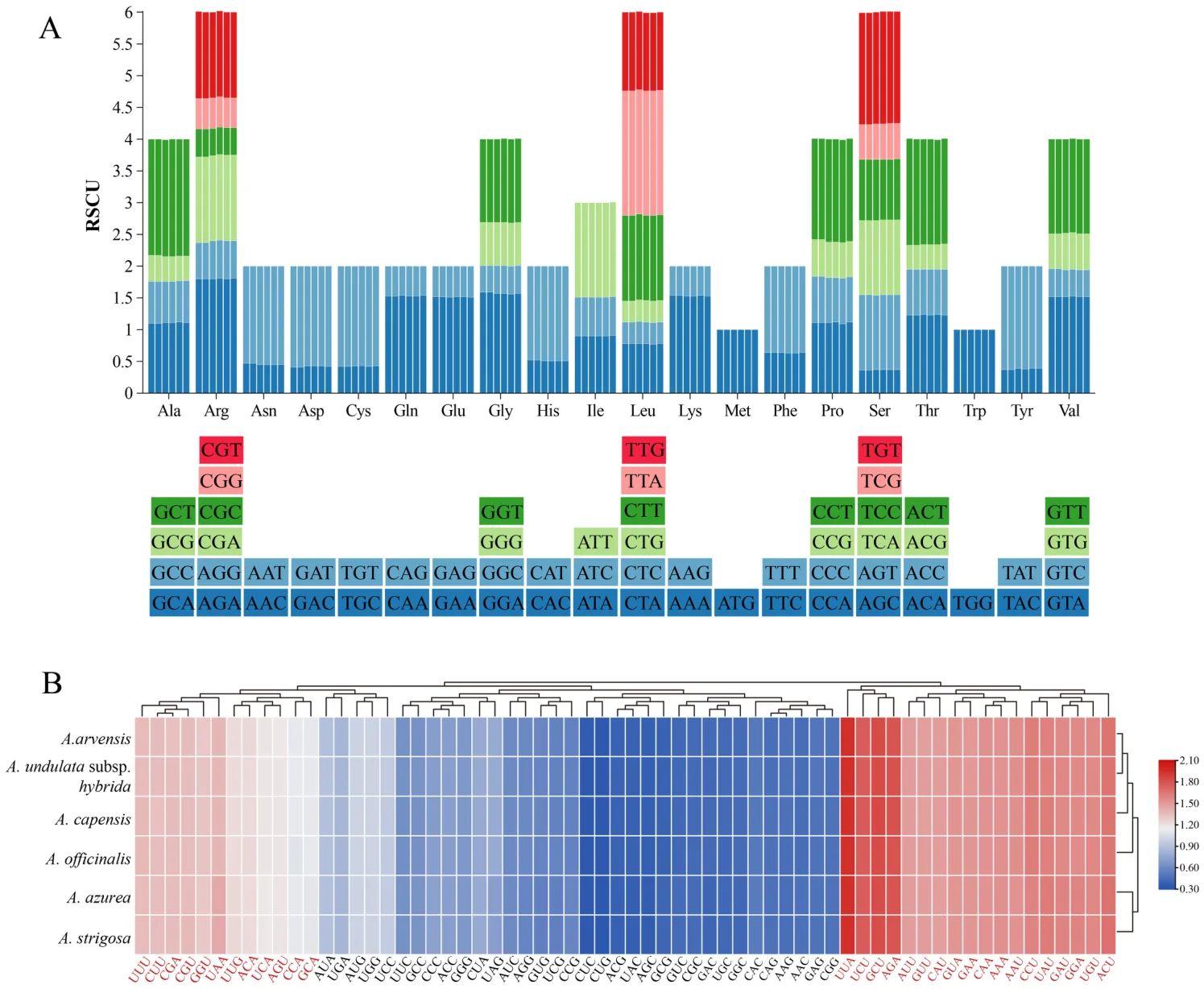
RSCU analysis and codon usage bias heatmap of six *Anchusa* species. (**A**) Bars for each amino acid are arranged from left to right as *A. strigosa*, *A. azurea*, *A. arvensis*, *A. capensis*, *A. officinalis*, *A. undulata* subsp. *hybrida.* The *x*-axis shows the 20 standard amino acids, and the *y*-axis shows the corresponding RSCU values. (**B**) Codon preference heatmap of six *Anchusa* chloroplast genomes; preferred codons are highlighted in red. Hierarchical clustering of *Anchusa* codon preference patterns is shown on the right.

RSCU analysis based on 50 shared CDSs revealed highly consistent codon usage patterns across the six species (Figure 4A). Each of the six sequences contained all 64 synonymous codons. Thirty codons exhibited positive usage bias (RSCU > 1). All of these ended in A or U, except for the leucine codon UUG (ending in G). This reflects a general preference for A/U-ending codons in the chloroplast genomes of this genus (Figure 4B). In contrast, 32 codons exhibited negative usage bias (RSCU < 1), of which 90.63% (29/32) ended in C or G. The methionine (AUG) and tryptophan (UGG) codons showed no apparent bias (RSCU = 1.00). The leucine codon UUA consistently displayed the highest RSCU value (~1.96) across all species, whereas the serine codon CUG showed the lowest (0.33–0.34). Kruskal–Wallis and Dunn’s test showed no significant differences in RSCU values among the six *Anchusa* species (*p* = 1.00; Supplementary Figure S1).

### 3.4. Comparative Analysis

**Figure 5:**
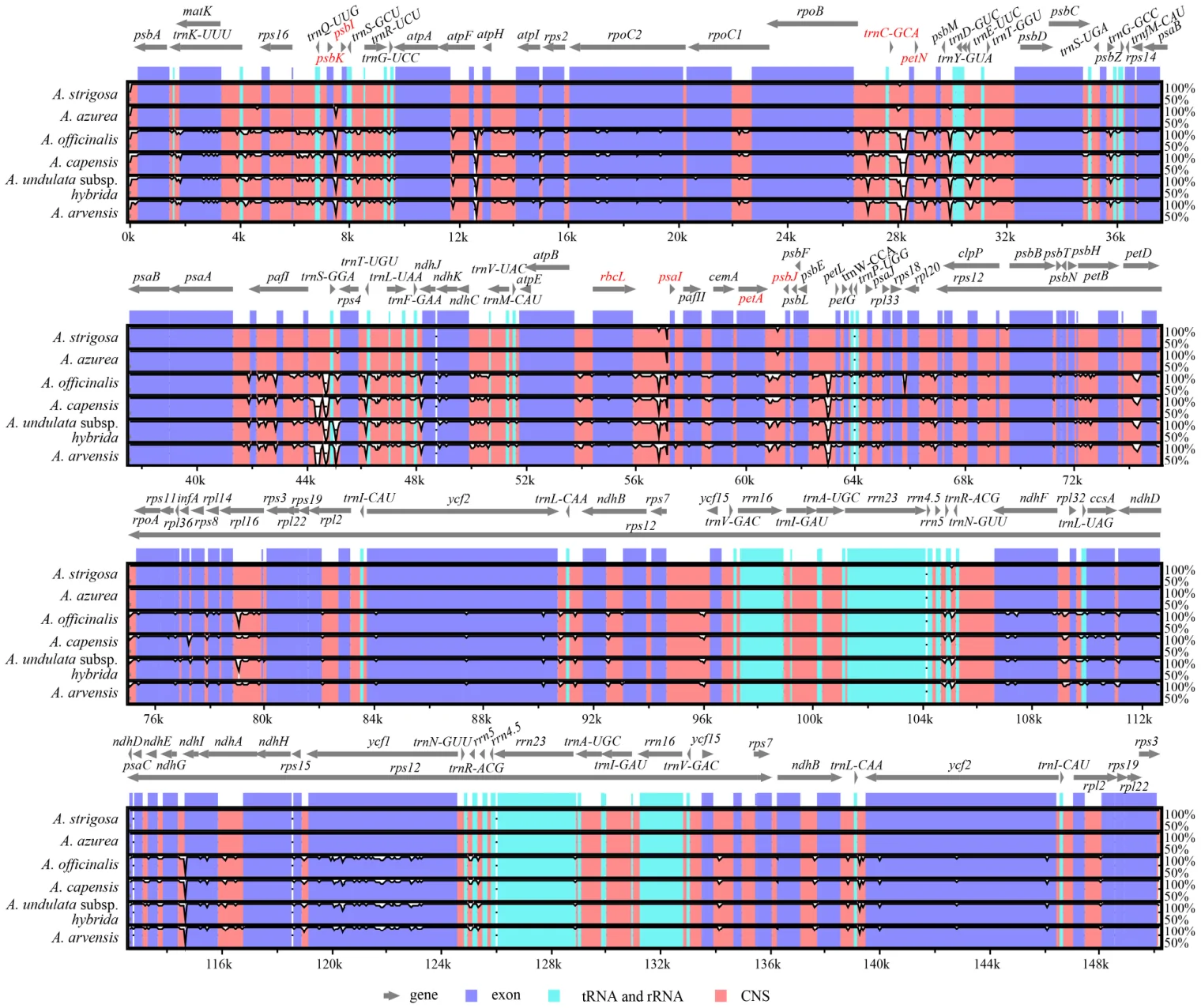
Visual alignment of chloroplast genome sequences of six *Anchusa* species (Candidate DNA barcode genes or intergenic spacer regions are highlighted in red).

Global alignment with mVISTA, using *A. strigosa* as the reference, revealed extremely high sequence conservation between *A. strigosa* and *A. azurea* within the same subgenus (subg. *Buglossum*). However, across all six species, the chloroplast genomes showed varying degrees of sequence divergence. Particularly pronounced differences were observed between phylogenetically distant subgenera. Four hypervariable regions were identified: *trnC-GCA*-*petN*, *psbK-psbI*, *rbcL*-*psaI*, and *petA*-*psbJ* (Figure 5). These regions showed clear divergence between subgenera and may serve as potential DNA barcodes or valuable molecular markers for phylogenetic studies.

**Figure 6:**
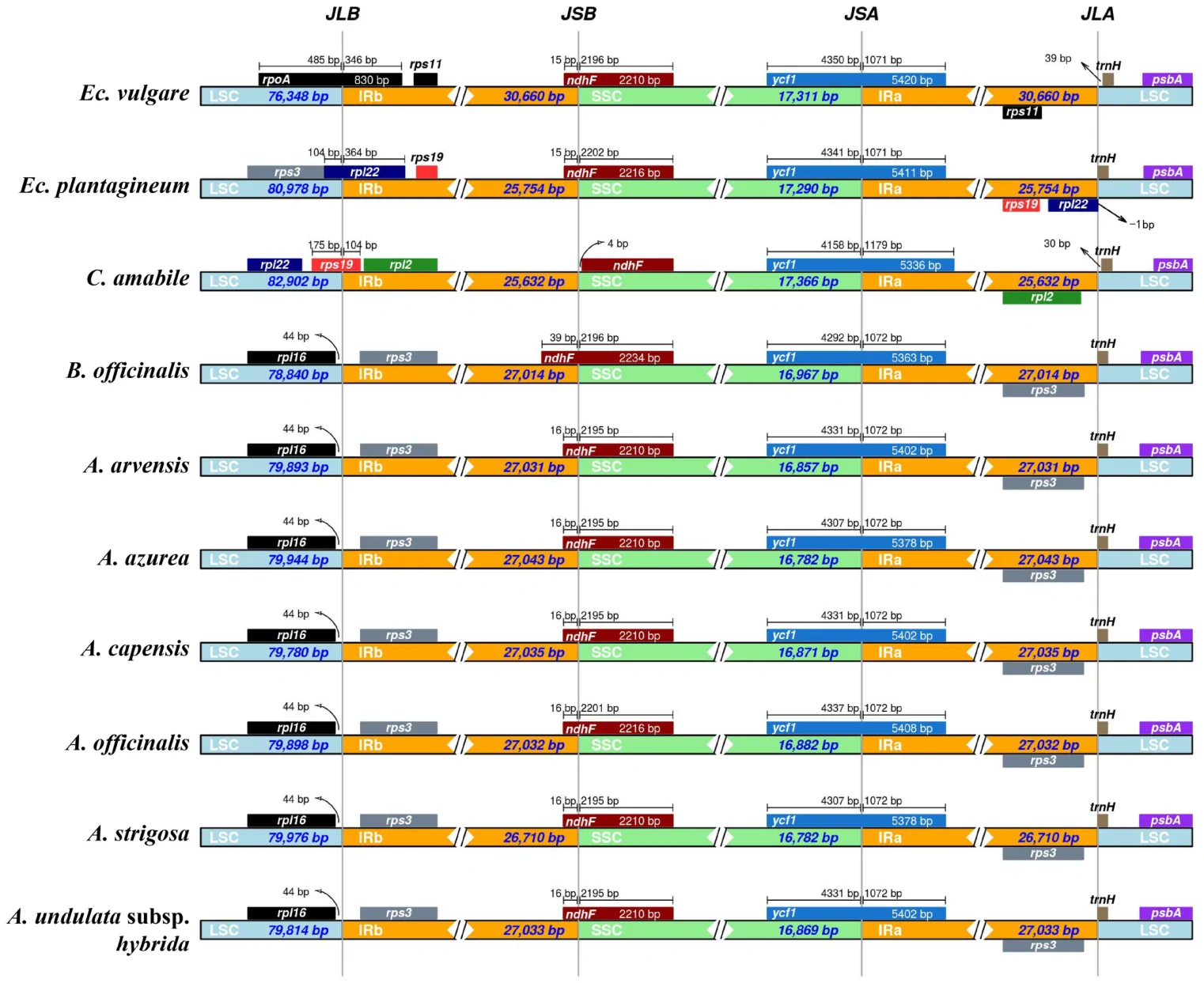
IR boundary analysis of *Anchusa* chloroplast genomes and related adulterants.

IRscope boundary analysis of IR, LSC, and SSC regions revealed highly conserved boundary structures among the six *Anchusa* chloroplast genomes (Figure 6). The boundary gene composition was identical across all species and included *rpl16*, *rps3*, *ndhF*, *ycf1*, *trnH*, and *psbA*. The *rpl16* gene consistently terminated 44 bp upstream of the LSC–IRb junction (JLB). The *ndhF* gene spanned the SSC–IRb junction (JSB) and extended 16 bp into the IRb region. The LSC, SSC, and IR region lengths were similar among species. No significant boundary expansion or contraction was observed.

The IR boundary gene composition of *B. officinalis* was identical to that of *Anchusa*, although its *ndhF* extended 23 bp farther into the IRb region (Figure 6). The IRa gene composition and JLB-proximal gene arrangement of *E. vulgare* and *C. amabile* differed markedly from those of *Anchusa* (Figure 6). In *C. amabile*, *rps19* extended from the LSC into the IRb region and resided at the JLB. Notably, *ndhF* did not extend into the IRb region in *C. amabile*. Within *Echium*, *E. vulgare* and its congener *E. plantagineum* differed in IRa gene composition and JLB-proximal gene arrangement. In *E. vulgare*, *rpoA* extended from the LSC into the IRb region. In *E. plantagineum*, *rpl22* extended from the LSC into the IRb region. The LSC–IRa boundary gene was *rps11* rather than *rps19*.

Collinearity analysis further confirmed the structural conservation of *Anchusa* chloroplast genomes, and all six genomes were collinear, with no detectable gene rearrangement events.

### 3.5. Nucleotide Diversity Analysis

**Figure 7:**
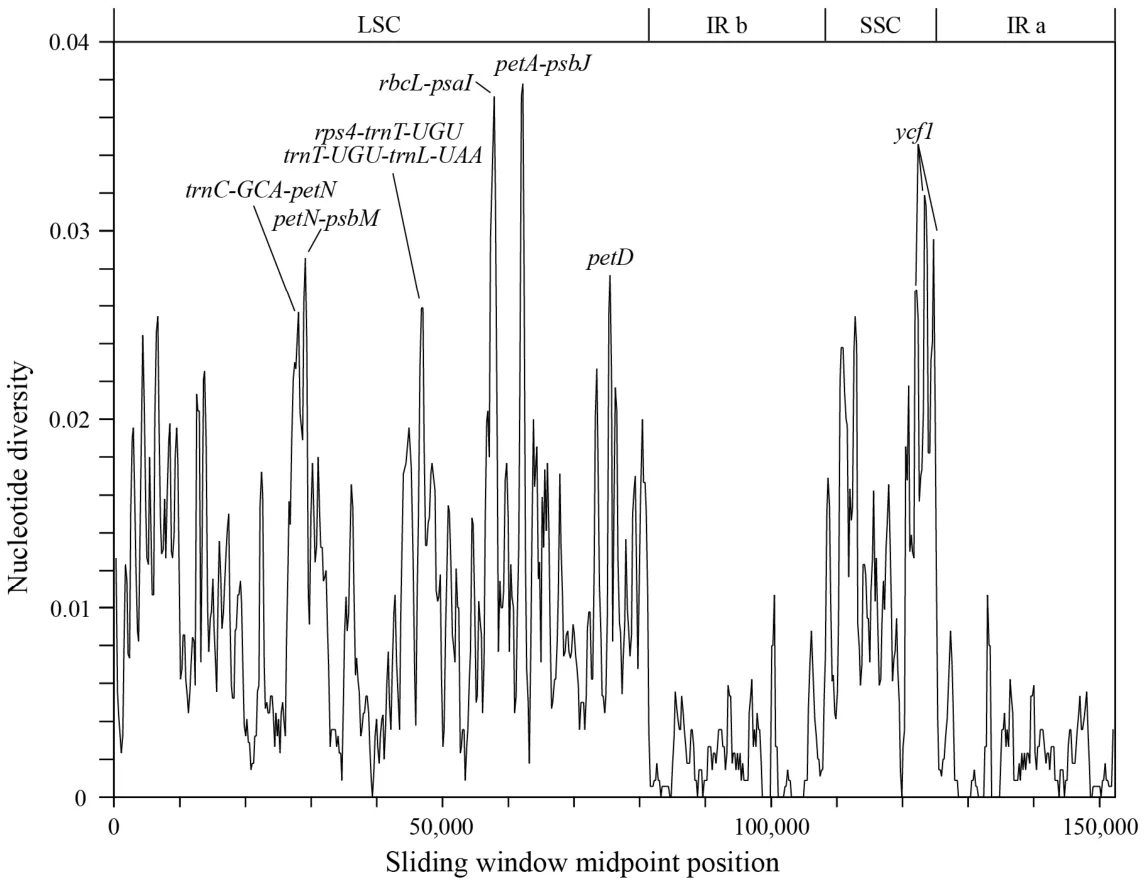
Sliding window analysis detecting hypervariable regions in *Anchusa* chloroplast genomes.

Sliding window analysis of the six *Anchusa* chloroplast genomes revealed nucleotide diversity (Pi) values ranging from 0 to 0.03778, with a mean Pi of 0.008129 (Figure 7). Eight fragments with Pi > 0.025 and length > 150 bp were identified as candidate sequences for species identification within *Anchusa* (Table 5). These included seven fragments in the LSC region (*petN-psbM*, *rbcL-psaI*, *petA-psbJ*, *trnC-GCA-petN*, *rps4-trnT-UGU*, *trnT-UGU-trnL-UAA*, *petD*) and one fragment in the SSC region (*ycf1*). Among them, *petA-psbJ* exhibited the highest Pi value (0.03778). Among these hypervariable regions, only one coding region (*ycf1*) showed elevated variability (Table 5). The remaining hypervariable fragments were located in intergenic spacer (IGS) regions. These hypervariable fragments may serve as candidate barcodes for species identification within *Anchusa*.

### 3.6. Phylogenetic Analysis

ML trees were constructed based on two datasets: complete chloroplast genomes and 78 shared CDSs from six *Anchusa* species, four adulterants *E. vulgare*, *E. plantagineum*, *B. officinalis*, and *C. amabile*, and three outgroup taxa.

**Figure 8:**
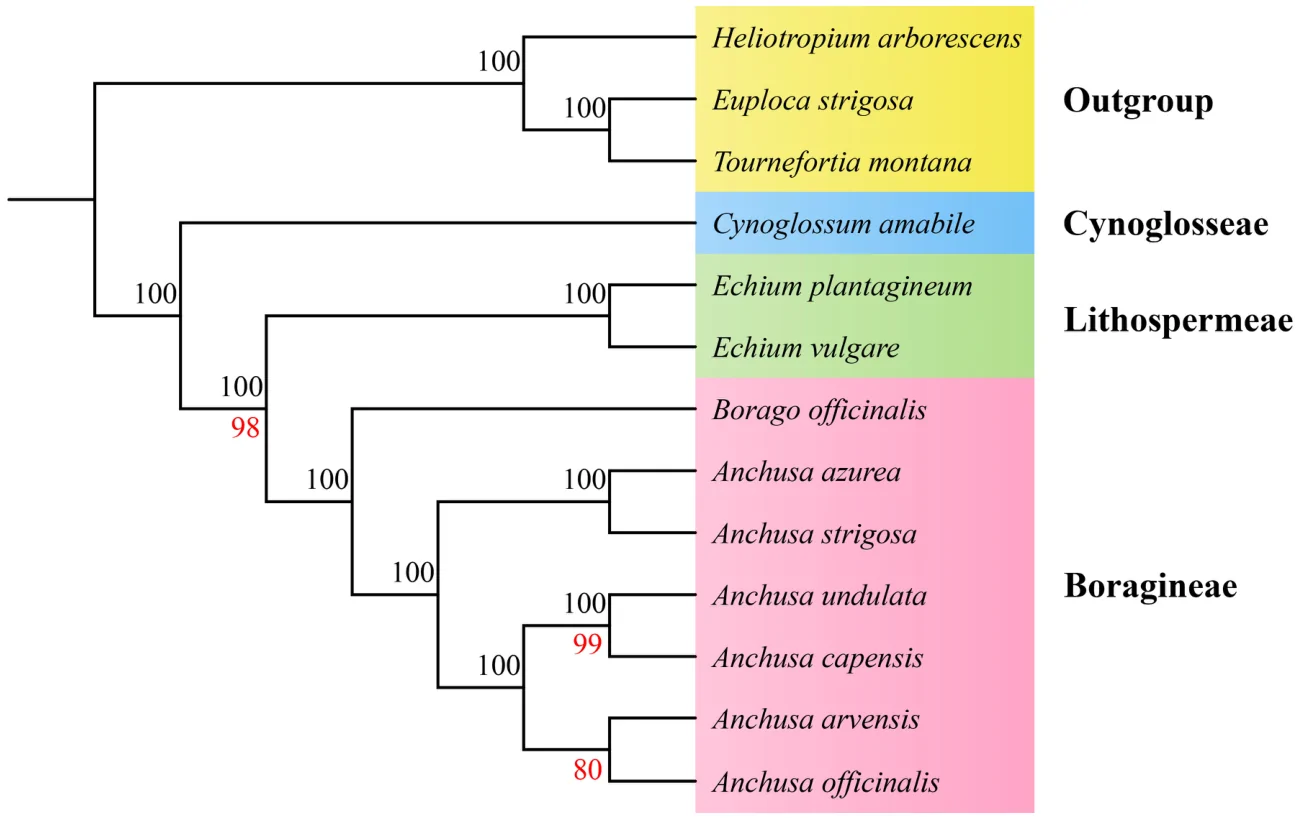
Maximum likelihood phylogenetic trees based on complete chloroplast genomes and CDS datasets (red numbers indicate bootstrap support (BS) values at nodes where the CDS-based tree differs from the complete chloroplast genome-based tree).

The two ML trees were topologically congruent (Figure 8). Differences in support values were observed only at some nodes. Nodes with lower support in the whole chloroplast genome tree obtained higher support in the CDS-based tree. *Anchusa* subg. *Anchusa* and *Anchusa* subg. *Buglossum* formed a clade (BS = 100%). This clade formed a sister group with *Borago* L. and constituted the tribe Boragineae (BS = 100%).

Within *Anchusa* subg. *Anchusa*, *A. capensis* was closely related to *A. undulata* subsp. *hybrida* (100% support in the whole chloroplast genome tree). *A. officinalis* was closely related to *A. arvensis* (80% support in the CDS-based tree).

## 4. Discussion

This study is the first comparative chloroplast genome analysis of six *Anchusa* species. The results not only elucidate the basic chloroplast genome features of the genus but also provide important molecular data for studies on origin identification and infrageneric phylogenetic relationships.

### 4.1. Conservation and Specificity of Anchusa Chloroplast Genomes

The six *Anchusa* chloroplast genomes were highly conserved. They showed similar genome size (150,178–150,844 bp), gene number (112), GC content (37.9%), and quadripartite structure. These features are consistent with those reported for most angiosperms [54]. Comparative genomic analysis revealed that *Anchusa* chloroplast genomes are highly similar overall. Single-copy regions (SSC) were more conserved than inverted repeat regions. Coding regions were more conserved than non-coding regions. Gene content, type, and arrangement were highly consistent across all species (Figure 5). IR/SC boundary gene compositions and positions were identical among all six species (Figure 6). The six sequences were collinear, and no recombination events were detectable. These findings indicate that the structure of the *Anchusa* chloroplast genome has remained stable during evolution. A similar pattern of conservation has been observed in other closely related groups, such as tribe Lithospermeae [55]. However, outside this lineage, as sequencing efforts have progressed, pervasive rearrangements have been revealed in plastid genomes across angiosperms, directly challenging the traditional assumption of a highly conserved structure [56]. This suggests that the absence of detectable rearrangements may represent a distinctive structural feature of the tribe Lithospermeae.

Nevertheless, structural conservation does not exclude sequence-level variation. Nucleotide diversity analysis identified eight hypervariable regions (Pi > 0.025), with *petA-psbJ* showing the highest variability (Pi = 0.03778). mVISTA-based visual comparison further identified four hypervariable regions: *trnC-GCA-petN*, *psbK-psbI*, *rbcL-psaI* and *petA-psbJ*. These regions are all located in intergenic spacers. They have accumulated greater variation and are therefore ideal candidates for DNA barcoding.

IR/SC boundary analysis revealed notable IR expansion in *E. vulgare* (Figure 6). Genes from SSC were incorporated into the IR regions. This changes its total gene number (including duplicated copies). The previously reported IR region of *E. nervosum* was only 19,204 bp, whereas the IR regions of *E. vulgare* and *E. plantagineum* were 30,660 and 25,754 bp, respectively. The three *Echium* species also exhibited different IR boundary gene compositions, suggesting considerable structural variation in the chloroplast genomes of the genus *Echium* [55].

### 4.2. Repeat Sequences and Codon Usage Bias

Repeat sequences in chloroplast genomes are an important source of evolutionary variation. They are rich in genetic information and serve as components of gene regulatory networks, regulating gene expression together with signaling molecules and cis-elements [57]. Analyzing repeat sequence distribution and abundance helps to understand genome stability, recombination hotspots, and potential evolutionary dynamics [58,59]. SSR loci are valuable for molecular marker development, species identification, population genetics, and phylogenetic studies due to their high polymorphism and codominant inheritance [60,61]. Therefore, this analysis of repeat sequences in six *Anchusa* chloroplast genomes provides key information for assessing genetic background, DNA barcode development, and the evolutionary history of this genus.

SSR analysis detected a total of 312 loci. Mononucleotide repeats consisting of A/T (65.31–73.68%), mainly distributed in the LSC region. This pattern is consistent with typical features of angiosperm chloroplast genome SSRs [62]. The variation in SSR numbers among species (49–57) provides a basis for future studies on intraspecific genetic diversity and species delimitation.

As shown in Figure 2, the total number of interspersed repeats in the six *Anchusa* species ranged from 44 to 51. Palindromic repeats were dominant, followed by forward repeats. In many angiosperms, palindromic repeats and forward repeats play key roles in maintaining chloroplast genome stability and regulating gene expression under abiotic stresses. They also promote adaptive evolution through homologous recombination. The genus *Anchusa* also shows a clear predominance of palindromic repeats and forward repeats. This pattern is consistent with the conserved mode observed across angiosperms. This balance reflects a trade-off between genome stability and adaptive variation facilitated by repetitive sequences [63]. Notably, the *Anchusa* chloroplast genomes have maintained high structural conservation during evolution. The collinearity analysis detected no rearrangement events within the genus. This is possibly because palindromic repeats primarily maintain structural stability rather than triggering large-scale rearrangements in *Anchusa* species.

Codon usage bias refers to the non-random selection of certain synonymous codons during translation. Although synonymous mutations do not alter the encoded amino acid sequence, growing evidence indicates that they play important roles in regulating gene expression and influencing protein structure. They are therefore not “silent” [64,65]. As previously reported, this bias results from natural selection, mutational pressure, and genetic drift over long-term evolution. This leads to variation among species and even among different genes within the same genome [66]. Generally, closely related species or those inhabiting similar environments tend to exhibit more similar codon usage patterns because of comparable selective pressures [67]. Therefore, comparative analysis of codon usage bias is important for understanding evolutionary patterns within *Anchusa*.

Analysis of the six *Anchusa* chloroplast genome sequences revealed that all 64 synonymous codons are present. Among these, 30 high-frequency codons (RSCU > 1) are identified. Except for UUG (ending in G), the remaining 29 high-frequency codons end in A or U. This indicates that codon usage in *Anchusa* chloroplast genomes is biased toward A/U-ending codons. This trend is consistent with findings in other angiosperms [55,68]. No significant differences in RSCU values were observed among the six *Anchusa* species (Supplementary Figure S1). This suggests that codon usage patterns are highly conserved within this genus.

### 4.3. Limitations of Conventional Barcodes and Identification of Specific Hypervariable Fragments

In plants, *matK* is considered one of the rapidly evolving coding regions. This is because all three codon positions evolve at nearly equal rates under relaxed purifying selection [69]. *rbcL* encodes the large subunit of RuBisCO [52]. It shows relatively low interspecific variability. Consequently, it is recommended for identification at the genus level and above. The *psbA-trnH* intergenic spacer has a high interspecific divergence rate and nearly 100% amplification success rate. Therefore, it has been widely used for species-level identification across diverse genera [70]. However, nucleotide diversity analysis revealed that three conventional barcodes (*matK*, *rbcL*, and *psbA-trnH*) are not the most variable markers within *Anchusa*. They show low nucleotide diversity (Pi = 0.01267–0.01956).

Sliding window analysis of the complete chloroplast genomes identified eight hypervariable fragments with high nucleotide diversity (Pi > 0.025) *(*Table 5). These include *petN-psbM*, *rb*c*L-psaI*, *petA-psbJ*, and y*cf1*, among others. Additionally, mVISTA-based comparison identified four hypervariable regions: *trnC-GCA*-*petN*, *psbK-psbI*, *rbcL-psaI*, and *petA-psbJ.* The hypervariable regions detected by the two methods partially overlapped, including *trnC-GCA*-*petN*, *rbcL*-*psaI* and *petA-psbJ*. Nevertheless, each method also identified exclusive regions (Table 5). This indicates differing thresholds for defining hypervariability.

mVISTA is based on global multiple alignment of complete sequences. One sequence is selected as the reference. Each query sequence is aligned to this reference. This approach preserves and visualizes large-scale insertions/deletions (indels). It displays percentage identity along the alignment [44]. In contrast, sliding window analysis excludes alignment gaps and computes average nucleotide diversity (Pi) through pairwise comparisons within non-overlapping windows [71]. That is, mVISTA quantifies sequence identity relative to a reference baseline. Nucleotide diversity (Pi) measures the mean pairwise divergence across all compared taxa. Considering the complementarity of the two methods, fragments shared by both approaches are proposed as more reliable candidates for DNA barcoding.

### 4.4. Phylogenetic Analysis

Phylogenetic trees were constructed using complete chloroplast genomes and the CDS datasets. They showed that all species of *Anchusa* subg. *Anchusa* formed a single clade (BS = 100%). All species of *Anchusa* subg. *Buglossum* formed another clade (BS = 100%). These two clades formed a sister group to the genus *Borago*. Within subg. *Anchusa*, *A. capensis* and *A. undulata* subsp. *hybrida* clustered as sister taxa (BS from whole chloroplast genome = 100%, BS from CDS = 99%). Similarly, *A. officinalis* and *A. arvensis* formed a sister pair (BS from CDS = 80%). These results provide a molecular framework for clarifying the relationships among morphologically similar species and are consistent with traditional classification based on morphological traits and with molecular phylogenetic studies using nuclear ITS and chloroplast *trnL* sequences [72,73].

However, our study did not include *Lycopsis* and *Cynoglotti*s, or the two subgenera *Buglossellum* and *Buglossoides*, which have been *s*egregated from *Anchusa* sensu lato. Therefore, the current phylogenetic tree cannot directly test or fully resolve the historically recognized paraphyly of *Anchusa* sensu lato.

## 5. Conclusions

The complete chloroplast genomes of six *Anchusa* species were compared. The chloroplast genomes of *Anchusa* are highly conserved in structure, exhibiting a typical quadripartite circular structure. Gene content, gene order, and IR/SC boundaries are consistent. No rearrangement events were detected. Sliding window analysis identified eight hypervariable fragments (Pi > 0.025). mVISTA analysis further identified four regions. Among these, *rbcL-psaI*, *petA-psbJ*, and *trnC-GCA-petN* were shared by both methods. These regions can serve as preferred candidate barcodes for species identification within *Anchusa*. Phylogenetic analyses confirmed that subg. *Anchusa* and subg. *Buglossum* each form a monophyletic clade. This result supports the taxonomic views based on morphological and molecular data.

In summary, this study elucidates the genetic structural characteristics and evolutionary relationships of *Anchusa* species at the chloroplast genome level. The hypervariable regions identified herein provide a solid foundation for developing efficient and accurate molecular identification methods. This will contribute to resolving the origin identification of Niushecao and provide new genomic evidence for taxonomic and systematic evolutionary studies of the genus.

However, several limitations should be acknowledged. First, each species was represented by a single sample without intraspecific replication. Therefore, the diagnostic power of the candidate markers at the species level could not be assessed. It is also difficult to distinguish intraspecific variation from interspecific divergence. Second, *A. azurea* was not sampled directly. Thus, the practical discrimination between the authentic medicinal material and its main adulterant, *A. strigosa*, could not be verified. Third, the phylogenetic analysis did not include *Lycopsis*, *Cynoglottis*, or the subg. *Buglossellum* and *Buglossoides*. Consequently, the paraphyly of *Anchusa* s. l. remains unresolved.

Future studies should expand sampling and include intraspecific replicates to validate marker stability. Additional evidence, such as chromosome data, should be incorporated. Nuclear genes should be integrated with chloroplast genome data for combined phylogenetic analyses. These efforts will further clarify the infrageneric classification and provide a robust taxonomic foundation for the medicinal and edible use of this genus.

## Figures and Tables

**Table 1:** Sample information.

Species	Sample Number	Collection Location and Time
*A. strigosa* Banks & Sol.	CL25102901	Jinghai Road, Hotan City, Hotan Prefecture, Xinjiang, 20251029
*E. vulgare* L.	CL25102101	Animal Husbandry Team, Sartam Township, Hababe County, Altay Prefecture, Xinjiang, 20251021

**Table 2:** List of studied species of *Anchusa* and relatives.

Family		Species	Accession Number
Boraginaceae	Boragineae	*A. strigosa*	PZ016459
		*A. azurea* Mill.	ERR14050913
		*Anchusa officinalis* L.	ERR14050915
		*Anchusa arvensis* (L.) M.Bieb.	OZ374795.1
		*Anchusa capensis* Thunb.	SRS21047483
		*Anchusa undulata* L. subsp. *hybrida* (Ten.) Cout.	ERR14050912
		*B. officinalis* L.	NC_046796.1
		*Nonea vesicaria* (L.) Rchb.	NC_060826.1
	Lithospermeae	*E. vulgare*	PZ233845
		*Echium plantagineum* L.	NC_067370.1
	Cynoglosseae	*C. amabile* Stapf & Drummond	NC_061706.1
Heliotropiaceae		*Heliotropium arborescens* L.	NC_066966.1
		*Euploca strigosa* (Willd.) Diane & Hilger	NC_084103.1
		*Tournefortia montana* Lour.	NC_066795.1

**Table 3:** Basic characteristics of the chloroplast genomes of *Anchusa* species and their adulterants.

Item	*A. strigosa*	*A. azurea*	*A. officinalis*	*A. capensis*	*A. arvensis*	*A. undulata* subsp. *hybrida*	*B. officinalis*	*E. vulgare*	*E. plantagineum*	*C. amabile*
Genome size (bp)	150,178	150,812	150,844	150,721	150,812	150,749	149,835	154,979	149,776	151,532
Length of LSC (bp)	79,976	79,944	79,898	79,780	79,893	79,814	78,840	76,348	80,978	82,902
GC content of LSC (%)	35.9	35.9	36.0	36.0	36.0	36.0	35.8	35.6	35.5	35.2
Length of SSC (bp)	16,782	16,782	16,882	16,871	16,857	16,869	16,967	17,311	17,290	17,366
GC content of SSC (%)	31.8	31.7	31.7	31.7	31.7	31.7	31.4	31.1	31	30.9
Length of IRs (bp)	26,710	27,043	27,032	27,035	27,031	27,033	27,014	30,660	25,754	25,632
GC content of IRs (%)	42.8	42.8	42.8	42.8	42.8	42.8	42.7	41.6	43	43.1
Total GC content (%)	37.9	37.90	37.9	37.90	37.90	37.90	37.80	37.40	37.50	37.40
Total number of genes	112	112	112	112	112	112	112	112	113	112
Number of protein-coding genes	78	78	78	78	78	78	78	78	79	78
Number of tRNA genes	30	30	30	30	30	30	30	30	30	30
Number of rRNA genes	4	4	4	4	4	4	4	4	4	4

**Table 4:** Gene composition of *Anchusa* chloroplast genomes.

Gene Function	Gene Category	Gene Name
Self-replication	Large subunit of ribosome protein	*rpl2* ^a,^* × 2, *rpl14*, *rpl16* *, *rpl20*, *rpl22* × 2, *rpl32*, *rpl*33, *rpl*36
	Small subunit of ribosome protein	*rps2*, *rps3* × 2, *rps4*, *rps7* ^a^ × 2, *rps8*, *rps11*, *rps12* ^a,b,^** × 2, *rps14*, *rps15*, *rps16* *, *rps18*, *rps19* ^a^ × 2
	Subunit RNA polymerase	*rpoA*, *rpoB*, *rpoC1* *, *rpoC2*
	Ribosomal RNAs	*rrn4.5* ^a^ × 2, *rrn5* ^a^ × 2, *rrn16* ^a^ × 2, *rrn23* ^a^ × 2
	Transfer RNAs	*trnA-UGC* ^a,^* × 2, *trnC-GCA*, *trnD-GUC*, *trnE-UUC*, *trnF-GAA*, *trnfM-CAU*, *trnG-UCC* *, *trnG-GCC*, *trnH-GUG*, *trnI-CAU* ^a^ × 2, *trnI-GAU* ^a,^* × 2, *trnK-UUU* *, *trnL-CAA* ^a^ × 2, *trnL-UAA* *, *trnL-UAG*, *trnM-CAU*, *trnN-GUU* ^a^ × 2, *trnP-UGG*, *trnQ-UUG*, *trnR-ACG* ^a^ × 2, *trnR-UCU*, *trnS-GCU*, *trnS-GGA*, *trnS-UGA*, *trnT-GGU*, *trnT-UGU*, *trnV-GAC* ^a^ × 2, *trnV-UAC* *, *trnW-CCA*, *trnY-GUA*
Photosynthesis	Subunit of photosystem I	*psaA*, *psaB*, *psaC*, *psaI*, *psaJ*, *pafI* **, *pafII*
	Subunit of photosystem II	*psbA*, *psbB*, *psbC*, *psbD*, *psbE*, *psbF*, *psbH*, *psbI*, *psbJ*, *psbK*, *psbL*, *psbM*, *psbN*, *psbT*, *psbZ*
	Subunit of NADH-dehydrogenase	*ndhA* *, *ndhB* ^a,^* × 2, *ndhC*, *ndhD*, *ndhE*, *ndhF* ^a^ *ndhG*, *ndhH*, *ndhI*, *ndhJ*, *ndhK*
	Subunit of cytochrome b/f complex	*petA*, *petB* *, *petD* *, *petG*, *petL*, *petN*
	Subunit of ATP synthase	*atpA*, *atpB*, *atpE*, *atpF* *, *atpH*, *atpI*
	RubisCO large subunit	*rbcL*
Other genes	Translation initiation factor	*infA*
	Maturase	*matK*
	Protease	*clpP* **
	Envelope membrane protein	*cemA*
	C-type cytochrome synthase	*ccsA*
Unknown function gene	Open reading frames	*ycf1*, *ycf2* ^a^ × 2, *ycf15* ^a^ × 2

^a^ Genes in inverted repeat regions; ^b^ trans-splicing gene; * genes containing one intron; ** genes containing two introns;  × 2 genes with duplicates.

**Table 5:** Information on hypervariable regions in *Anchusa* chloroplast genomes.

No.	Fragment	Fragment Length/bp	Nucleotide Diversity (Pi)	Region
1	*trnC-GCA-petN*	629–822	0.02567	LSC
2	*petN-psbM*	854~893	0.02856	LSC
3	*rps4-trnT-UGU*	312~353	0.02589	LSC
4	*trnT-UGU-trnL-UAA*	703~705	0.02589	LSC
5	*rbcL-psaI*	1362~1727	0.03711	LSC
6	*petA-psbJ*	809~825	0.03778	LSC
7	*petD*	1218~1231	0.02767	LSC
8	*ycf1*	5379~5403	0.03189	SSC

## Data Availability

The chloroplast genome sequences have been deposited in GenBank under accession numbers PZ016459 (*A. strigosa*), PZ233845 (*E. vulgare*). The raw sequencing data supporting these assemblies have been deposited in the NCBI Sequence Read Archive (SRA) under BioProject PRJNA1502504. The publicly available datasets analyzed in this study were obtained from GenBank, ENA, and SRA repositories. A complete list of all accession numbers and their corresponding species is provided in Table 2.

## Supplementary Materials

The following supporting information can be downloaded at: https://www.mdpi.com/article/10.3390/genes17090993/s1, Supplementary Figure S1. Boxplot of Kruskal–Wallis/Dunn test results for RSCU among six Anchusa.

## Abbreviations

The following abbreviations are used in this manuscript:

BS	Bootstrap support
CDS	Coding DNA Sequence
GUCM	Guangzhou University of Chinese Medicine
IGS	Intergenic spacer
IR	Inverted repeat
JLB	LSC–IRb junction
JSB	SSC–IRb junction
LSC	Large single-copy
ML	Maximum likelihood
RSCU	Relative synonymous codon usage
SSC	Small single-copy
SSRs	Simple Sequence Repeats
