# Supplementary material for: Comparative Chloroplast Genome Analysis of Anchusa and the Adulterants of HERBA ANCHUSAE
Source: Genes (Basel). 2026 Aug 24;17(9):993. doi: 10.3390/genes17090993 (PMC13606291; doi:10.3390/genes17090993)
Supplement: Supplementary file 1 [file genes-17-00993-s001.zip › genes-4365857-supplementary.pdf]

genes

MDPI

Article

Comparative Chloroplast Genome Analysis of Anchusa and the Adulterants of HERBA ANCHUSAE

Supplementary Materials

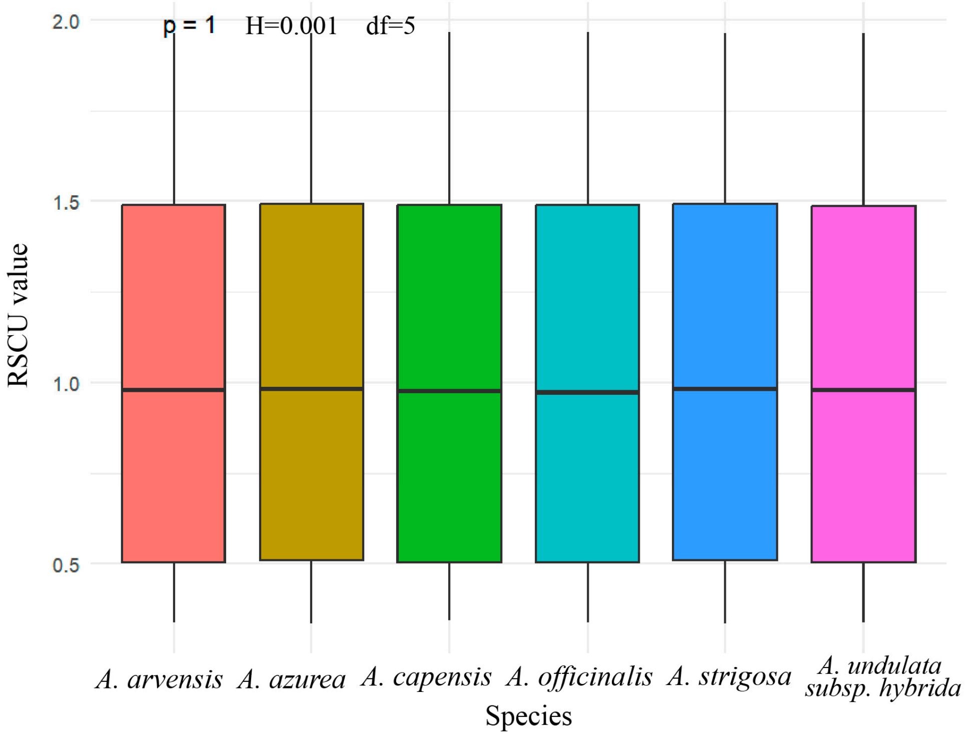

Supplementary Figure S1. Boxplot of Kruskal–Wallis/Dunn test results for RSCU among six *Anchusa*.

 https://doi.org/10.3390/genes17090993
